# Assessment of Biological Contribution to Natural Recovery of Anthropized Freshwater Sediments From Argentina: Autochthonous Microbiome Structure and Functional Prediction

**DOI:** 10.3389/fmicb.2021.601705

**Published:** 2021-03-05

**Authors:** Laura Madueño, Viviana Ayelen Starevich, Ana Carolina Agnello, Bibiana Marina Coppotelli, Cecilia Laprida, Nuria Carolina Vidal, Pablo Di Marco, Maria Elena Oneto, Maria Teresa Del Panno, Irma Susana Morelli

**Affiliations:** ^1^CINDEFI, UNLP-CONICET, La Plata, Argentina; ^2^Instituto de Estudios Andinos, CONICET/UBA, Ciudad Autónoma de Buenos Aires, Argentina; ^3^YPF-Tecnología, La Plata, Argentina; ^4^YPF S.A., Buenos Aires, Argentina; ^5^CIC-PBA, La Plata, Argentina

**Keywords:** monitored natural attenuation, bioremediation, fresh-water sediments, hydrocarbons, Buenos Aires, metabarcoding, functional prediction

## Abstract

Monitored natural recovery (MNR) is an *in situ* technique of conventional remediation for the treatment of contaminated sediments that relies on natural processes to reduce the bioavailability or toxicity of contaminants. Metabarcoding and bioinformatics approaches to infer functional prediction were applied in bottom sediments of a tributary drainage channel of Río de La Plata estuary, in order to assess the biological contribution to MNR. Hydrocarbon concentration in water samples and surface sediments was below the detection limit. Surface sediments were represented with high available phosphorous, alkaline pH, and the bacterial classes Anaerolineae, Planctomycetia, and Deltaproteobacteria. The functional prediction in surface sediments showed an increase of metabolic activity, carbon fixation, methanogenesis, and synergistic relationships between Archaeas, Syntrophobacterales, and Desulfobacterales. The prediction in non-surface sediments suggested the capacity to respond to different kinds of environmental stresses (oxidative, osmotic, heat, acid pH, and heavy metals), predicted mostly in Lactobacillales order, and the capacity of Alphaproteobacteria, Betaproteobacteria, Gammaproteobacteria, and Actinomyces classes to degrade xenobiotic compounds. Canonical correspondence analysis (CCA) suggests that depth, phosphate content, redox potential, and pH were the variables that structured the bacterial community and not the hydrocarbons. The characterization of sediments by metabarcoding and functional prediction approaches, allowed to assess how the microbial activity would contribute to the recovery of the site.

## Introduction

Petroleum exploitation in Argentina began in 1907 with the discovery of crude oil in the region of Patagonia. Throughout the 20th century, it registered a sustained growth that was accompanied by the installation of refineries to supply the domestic market and even export oil by products. The crude oil produced in Patagonia is transported to the refineries by trunk lines and sea. Basins have a local pipeline network that carries the extracted crude to the loading terminals of ships in order to transport it mainly to the operating refineries of Buenos Aires region (Riccardi, 2015). It is not uncommon that the exploitation and transportation of the crude oil generate and discharge into aquatic environments. When hydrocarbons are released into water courses, they are scavenged from the water column to the bottom through a number of processes, such as flocculation, sedimentation, and/or coagulation. This leads to concentrations in the sediment many orders of magnitude higher than in the water column, being the sediments, therefore, a potential sink for petroleum hydrocarbons (Adeniji et al., 2017). Once on the sedimentary column, hydrocarbons suffer from a wide variety of processes including: dilution, volatilization, chemical transformation, and biodegradation (Rothermich et al., 2002; Magar and Wenning, 2006; Morasch et al., 2007), as well as solid-association with sediments (Bao et al., 2013). Besides, another possible fate is the migration through the dynamic environment to other compartments, ultimately being propagated through the food chain (Xia et al., 2012).

One of the conventional remediation options for the treatment of contaminated sediments is the monitored natural recovery. MNR is an *in situ* approach that relies on biological, chemical, and physical natural processes that contribute to reducing the concentration, mobility, and/or toxicity of the contaminant (Foote et al., 2014). Biological and chemical processes can transform and remove contaminants. In addition, physical processes, like the deposition of fresh and clean sediment can bury and confine contaminated sediments through the formation of a natural cap. Physical processes typically occur faster than chemical and biological transformations (Lin et al., 2017), but altogether the time required for risk reduction can be relatively long. On the other hand, one of the main implications of the use of natural processes to reduce risks is the absence of building costs, often associated with other technologies.

Before selecting MNR as the remediation strategy, several lines of evidence are required in order to identify and evaluate the processes that could effectively contribute to pollutant risk reduction. For this purpose, is essential a detailed characterization involving biological, chemical, and physical analyses that support the contaminant source control, the chemical degradation, and the occurrence of physical processes. Likewise, biological trials that provide the proof of biodegradation are also essential. It results from the above that the application of MNR is the result of a thoughtful decision-making process following careful site assessment and characterization (National Research Council, 2001; US Environmental Protection Agency, 2005; Magar and Wenning, 2006).

Sediments of lakes, rivers, streams, and ponds are environments with a high microbial diversity. Bacteria, archaea, and eukaryotic microorganisms play important roles in natural biogeochemical processes, like organic matter decomposition, nutrient cycling, and food webs (Gadd and Raven, 2010; Pernthaler, 2013). Particularly, bacteria influence several processes, such as the consumption of oxygen and the turnover of nutrients (Tšertova et al., 2013). Microbes have the adaptive advantage of using hydrocarbons as an optional energy source in hydrocarbon contaminated ecosystems, regulating the nutrient flux with their surrounding environment. However, microbial dynamics in association with hydrocarbon contaminated environments are still poorly studied (Reid et al., 2018).

Recently, the development of environmental DNA metabarcoding techniques have revolutionized modern biodiversity surveys increasing the confidence and the potential of biomonitoring programs in the risk assessment of pollutants (Goodwin et al., 2017; Wang et al., 2020). Although information derived from DNA sequencing is yet to be formally included in assessment programs (Bourlat et al., 2013; Aylagas et al., 2020), nowadays this methodology is receiving major attention, and several projects start to involve evaluations based in metabarcoding techniques and bioinformatics approaches (Goodwin et al., 2017; Mukherjee et al., 2017; Aylagas et al., 2020). Sequencing studies generating data on autochthonous microbial communities are vital to develop ecosystem management programs and to design efficient clean-up strategies to cope with pollutants.

Before selecting MNR as an option for site management is necessary to provide evidence that natural remediation processes are occurring. As a result, the present work was conducted with the aim to establish the microbiological processes that could be involved in an ecosystem from a tributary drainage channel of Río de La Plata estuary. The central hypothesis of this work is that the study of the diversity and functional potential of sediment bacterial community using metabarcoding and bioinformatics tools as well as its interpretation considering a detailed physicochemical and geochemical characterization of the site, will allow to identify the hydrocarbon bioremediation potential and support the selection of MNR recovery strategy.

## Materials and Methods

### Sampling Sites

The selected site was a tributary drainage channel of Río de La Plata estuary. Several industries, mainly petrochemicals and oil refineries, are located at both sides of this channel, close to its mouth and in a relatively small area. While discharges occurred in the past, for the last 20 years, no new wastes or discharges were created. Sediment cores and water samples were collected in February 2017 at four sites along this area near of Río de la Plata estuary. During sampling, two active urban discharges were detected on the left margin of the stream. Sampling and description of sediment cores was carried out by the Laboratory of Continental and Marine Cores (SACMa, Instituto de Estudios Andinos Don Pablo Groeber, UBA CONICET, Argentina). Undisturbed cores were retrieved from the selected sampling sites from a platform with a manually operated hammer corer with transparent PVC liners devoid of core catcher. The single core tubes measured up to 2,000 mm in length with an internal diameter of 68 mm. The coring procedure was the same for all sampling points: the corer was lowered into the sediment where it penetrated initially by its own weight. Thereafter, a driving weight (the “hammer”) was lifted from the top of the corer and then released to drive the core tube into the sediment. Visual inspection after sampling allowed confirming that the water–sediment interface was kept at an undisturbed state in the liner, even after the corer was lifted to the surface.

Because of the highly fluid near-surface sediments, the core was transported from the field in the core tube and split in the laboratory by means of a longitudinal section.

Core surfaces were then cleaned, properly oriented, and measured. Lithology was visually described considering a number of parameters: the nature of the sediment, grain size, wet and dry coloring according to Munsell Color (Org; 2000), sedimentary structure, water content, and the presence of biogenic material. Any unusual features were noted. Major horizons or lithological units were defined for each core. Sediment subsamples were taken out from each horizon immediately after core opening. Samples were named according to the core number (1–4) and the horizon (H), being H0 the uppermost unit defined, i.e., the top core, and H6 the deepest (Supplementary Table S1). Samples were collected from the middle part of each horizon avoiding the contact between adjacent units but also the tube wall. This was particularly important to avoid the potential dragged-in contamination due to the plastic deformation of the sediment column. Samples were collected in sterile glass bottles or polypropylene tubes. According to lithological units, a total of 22 sediment subsamples were taken for subsequent analysis (Supplementary Table S1). Samples were then stored at −20°C in order to perform DNA extraction.

### Sediment Physicochemical Analyses

The external laboratory INDUSER S.A. performed physicochemical analyses in water and sediment samples. Polycyclic aromatic hydrocarbons (PAH; EPA 3535 A/8310), benzene, toluene, ethylbenzene, and xylene (BTEX; EPA 5021 A/8260 C), aliphatic hydrocarbons C6-C35 (TNRCC 1006), aromatic hydrocarbons C7-C35 (TNRCC 1006), total hydrocarbons (THC) C6-C35 (TNRCC 1005), pH (EPA 9045 D), and redox potential [SM 2580 Ed. 22 (#)] were determined in water and sediment samples. Total nitrogen (SM 4500 to N org C/NH3 C), soluble sulfate [USDA LMM 4D2a2/SM 4110 B(#)], total sulfide (EPA 9030 B/9034), available phosphorous (SM 2580 or Acid digestion/SM 4500-P C/E), exchangeable ammonium (NOM-021-RECNAT-2000 AS-08), pH [SM 4500-H B Ed. 22 (#)], organic matter (NOM-021-RECNAT-2000 AS-07), ion exchange capacity (NOM-021-RECNAT-2000 AS-12/13), water content (SM 2540 G), the ratios of nC17/pristane and nC18/phytane (EPA 3585 A/8015 C), and heavy metals like arsenic, cadmium, total chromium, and lead (EPA 3015 A/6020 B) were measured in sediment samples.

### DNA Extraction, 16S rRNA Gene Amplification, and Illumina MiSeq Sequencing

The DNA of sediment samples was extracted using E.Z.N.A Soil DNA kit (OMEGA, United States) according to the manufacturer protocol and stored at −20°C until further analysis. DNA extracts were checked for quantity and purity with NanoDrop™ 2000/2000c Spectrophotometers (Thermo Fisher Scientific) and subsequently submitted to the sequencing platform Mr. DNA (Molecular Research LP, Shallowater, TX). The 16S ribosomal RNA (rRNA) gene V4 variable region PCR primers 515/806 for Eubacteria and Archaea with barcode on the forward primer were used in a 30 cycle PCR using the HotStarTaq Plus Master Mix Kit (Qiagen, United States) under the following conditions: 94°C for 3 min, followed by 28 cycles of 94°C for 30 s, 53°C for 40 s, and 72°C for 1 min, after which a final elongation step at 72°C for 5 min was performed. After amplification, PCR products were checked in 2% agarose gel to determine the success of amplification and the relative intensity of bands. PCR products were used to prepare a DNA library following Illumina TruSeq DNA library preparation protocol. Sequencing was performed on Illumina MiSeq Next Generation Sequencer in accordance with the manufacturer guidelines.

### 16S rRNA Amplicon Processing and Bioinformatic Analysis

The sequences generated in the present study were deposited in the National Center of biotechnology information^1^ as the Bioproject “Natural recovery of anthropized fresh-water sediments”, and are available under the accesion number PRJNA592525.

The raw data (FastQ files) deposited in a cloud-based platform (BaseSpace™) were demultiplexed by FastQ Processor tool (MR DNA free software application). Paired reads were joined and sequences were analyzed following the 16S Bacteria and Archaea Standard Operating Procedure available through the Microbiome Helper website and fully described by Comeau et al. (2017). Briefly, the pipeline performed a quality control of raw paired-end reads with FastQC (v0.11.5; Andrews, 2010) and stich the unambiguous forward and reverse sequences using PEAR (v0.9.10; Zhang et al., 2014). Then, sequences with quality score less than 30 over at least 90% of the bases, shorter than 250 bp and with unknown bases, were eliminated using FASTX-Toolkit (v0.0.14; Gordon, 2009). The chimeric sequences were removed using VSEARCH (v1.11.1; Rognes et al., 2016), which implements the UCHIME algorithm (Edgar et al., 2011) and grouped into different operational taxonomic units (OTUs; at 97% identity) performing open-reference OTU picking with QIIME 1 (v1.9.1; Caporaso et al., 2012; Rideout et al., 2014). Finally, singletons and low-confidence OTUs were eliminated. Open-source methods SortMeRNA (v2.0; Kopylova et al., 2012) and SUMACLUST (v1.0.00; Mercier et al., 2013) were used for the reference-based and *de novo* clustering steps, respectively. The taxonomic assignment was carried out using the Greengenes database (McDonald et al., 2012), filtering out OTUs having <0.1% of the total number of sequences. Subsequently, the table of OTUs was normalized with the minimum number of reads per sample (20,259) and used to perform rarefaction curves, calculate diversity indices (Good, Shannon, Chao, Simpson), and estimate beta-diversity running principal-coordinate analysis (PCoA) on UniFrac distances (Lozupone et al., 2011; Vázquez-Baeza et al., 2013).

### Statistics

Differences in diversity indexes and bacteria relative abundances among sediment samples were compared *via* statistical ANOVA and *post hoc* Tukey-Kramer tests, using online calculator SciStatCalc version 1.4^2^ and the Statistical Analysis of Metagenomic Profiles^3^ (STAMP v2.1.3) software (Parks et al., 2014), respectively. Additionally, multiple test correction Benjamini-Hochberg FDR with a *q* value filter of 0.05 was applied in bacteria relative abundances using STAMP software. The differences in bacterial community structure among samples based on weighted UniFrac distance metric in PCoA were assessed through the PERMANOVA test with 999 permutations in QIIME 1.

### Canonical Correspondence Analysis

The data of physicochemical analysis and taxonomic assignment were used for multivariate analysis. Canonical correspondence analysis (CCA) between microbial diversity (Species data) and physicochemical parameters (Environment data) was performed using the software Canoco 4.5 (Microcomputer Power, Ithaca, NY, United States) in association with CanoDraw®. Relative abundances (x) at class level (Species data) were transformed by arcsin (x/100)^1/2^, while the environmental variables were transformed using log_10_(*x* + 1). Chloroplast, unassigned sequences, and classes below 5% of relative abundance were removed for the species data matrix for further analysis. To obtain tests of independent effects of physicochemical variables, co-variables were eliminated in CCA. The effect of each environmental variable and their interaction on the sum of all canonical eigenvalues was tested by Monte Carlo permutation tests available in Canoco using 999 replicate runs to assess significance on the trace values.

### PICRUSt Functional Prediction

PICRUSt (v1.1.0) was used to infer the functional potential of prokaryotic communities in terms of Kyoto Encyclopedia of Genes and Genomes^4^ (KEGG) orthology and pathways, and to associate taxonomic changes with functional differences (Langille et al., 2013). The OTU table was used as the input file for the metagenome prediction. Major steps for the bioinformatics protocol include: (i) reducing the OTU table to just the reference OTUs, (ii) normalizing the OTU table by predicted 16S rRNA copy numbers, (iii) predicting abundances of KEGG orthology (K0s) in each sample, (iv) collapsing K0s into KEGG Pathways defined at levels 1–3 (Ogata et al., 1999; Kanehisa and Goto, 2000), and (v) connecting the OTUs that are contributing to each K0 of interest. STAMP (v2.1.3) was used for data visualization and statistical analyses (Parks et al., 2014). The Nearest Sequenced Taxon Index (NSTI) score, which is the sum of phylogenetic distances for each OTU between its nearest relative with a sequenced reference genome, measured in terms of substitutions per site in the 16S rRNA gene and weighted according to the frequency of that OTU, was used as an indicator for the accuracy of PICRUSt.

## Results

### Core Description

**Figure 1:**
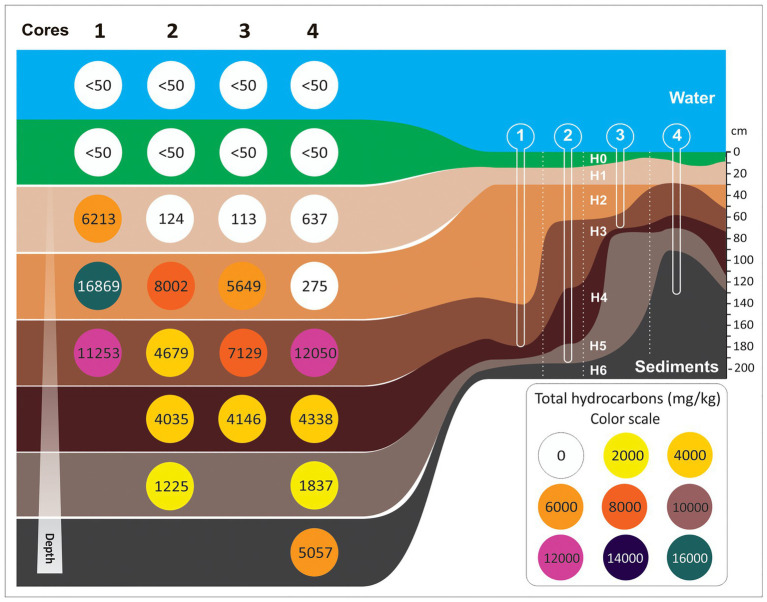
Schematic representation of the horizons length (cm) and hydrocarbon concentration (mg/kg) on each core along the freshwater channel of this study.

Sediment cores showed negligible shortening and no evidence of vertical mixing. Water–sediment interface and top layers of sediment were conveniently preserved. Core lengths varied between 74.5 cm and 194 cm, and the number of lithological units/horizons described varied between 3 and 7. Notably, the number of horizons recognized does not necessarily correlate with core length (Figure 1).

Cores 1 and 2 showed similar stratigraphic sequences. According to visual inspection, the topmost part of the sediment column was composed of massive rich organic muds. The color of these cores was black or dark greenish gray when the sample was moist and turned to olive/greenish-gray after drying. Toward the base, the organic matter rich layers rich organic mud layers were interbedded with centimeter-scale layers of hydrocarbon-rich muds. Dark gray, black, and bluish-black, faint-laminated hydrocarbon-rich muds underlie these levels. The base of the cores was composed of laminated light gray silty clays with fragments of molluscs valves.

Cores 3 and 4 were stratigraphically more complex. They were composed of several horizons of hydrocarbon-rich mud layers evident because of differences in color, consistency, and proportions of silt and clay. These horizons usually presented abundant, well-preserved particulate organic matter composed mainly by plant fragments, rubble, and debris derived from domestic activities. In core 4, a carbon-rich, semi-consolidated horizon, probably derived from oil refining, was recognized. The base of the sequence of core 4 was characterized by fine-grained sediments, mainly dark gray clayed silt with faint lamination and some discrete levels of sandy silt. The core length and the number of horizons of each core are summarized in Supplementary Table S1.

### Physicochemical Characteristics of the Sediments

The physical and chemical characteristics [i.e., PAH, BTEX, aliphatic and aromatic hydrocarbons, ratios nC17/pristane (nC17/Pr), nC18/phytane (nC17/Ph), total nitrogen, soluble sulfate, total sulfide, available phosphorus, pH, redox potential, organic matter, cation exchange capacity, humidity, and heavy metals] that were studied in sediment samples are summarized in Table 1. From these data, it can be seen that, independently of the core, the lowest values of hydrocarbons (i.e., PAH, BTEX, aliphatic hydrocarbons, aromatic hydrocarbons, total hydrocarbons) were found in samples from surface horizons (1H0, 2H0, 3H0, and 4H0). Also, most of sediment samples presented values of the nC17/Pr and nC18/Ph ratios lower than 1 (0.01–0.47) and (0.02–0.79), respectively. The ratios of surface samples 1H0, 2H0, and 4H0 were not possible to calculate due to nC17 and nC18 were below to detection limit (<50 mg/kg). pH was within the range 6.7–8.3, with the exception of samples from horizons H1, H2, H3, and H4 from core 4 (in the point of the urban discharge), in which pH values were downright acid (3.0–4.1). Redox potential was in average 200 mV for all the sediments, being indicative of a rather oxidizing environment. The content of organic matter was higher in middle sediments compared to surface and base sediments. Total nitrogen values were in average 1,500 mg/kg for sediments samples, with the highest value in 4H2 (2,610 mg/kg). Both soluble sulfate and total sulfide tended to increase with core depth. However, total sulfide was below the detection limit (50 mg/kg) in surface sediments of cores 1 and 2 and in almost sediments samples from core 4. Available phosphorus showed the highest values in H0 and H1 sediments of core 3 (52.3 and 58.2 mg/kg), while in the other samples it was found an average of 8 mg/kg. Arsenic, cadmium, chromium, and lead, were below the permitted sediment values according to the Interim Sediment Quality Guideline [ISQG from Canadian Environmental Quality Guideline (CEQG); 5.9, 0.6, 37.3, and 35 mg/kg, respectively; data not shown].

**Table 1:** Physicochemical characteristics in the sediments and water samples.

CORES	Parameters		PAH	BTEX	Aliphatic hydrocarbons	Aromatic hydrocarbons	Ratio nC17/pristane	Ratio nC18/phytane	Total nitrogen	Soluble sulfate	Total sulfide	Available phosphorus	pH	Redox potential	Exchangeable amonnium	Organic matter	Ionic exchange capacity	Water content
	Units		mg/kg	mg/kg	mg/kg	mg/kg	–	–	mg/kg	mmol/l	mg/kg	mg/kg	UpH	mV	mg/kg	% p/p	Cmol(+)/kg	% p/p	
1	water		nd	nd	ND	ND	ND	ND	ND	ND	ND	ND	9	304	ND	ND	ND	N
	sediments	H0	1.3	0.05	nd	nd	ND^*^	ND^*^	1215	0.63	nd	15.1	8.1	176	96.8	15.8	31.5	15.8	
		H1	9.7	1.805	2759	2446	0.03	0.02	1580	0.93	143	13.1	7.9	187	165	37.7	21.6	37.7	
		H2	12.3	45.69	7978	6529	0.03	0.160	1790	1.19	652	20.6	7.8	179	250	44.2	12.3	44.2	
		H3	16.7	0.05	5479	4131	0.03	0.12	1055	2.95	292	nd2	8.3	200	8.24	18	17.7	18	
2	water		nd	nd	ND	ND	ND	ND	ND	ND	ND	ND	9.3	303	ND	ND	ND	NC
	sediments	H0	0.2	0.05	nd	nd	ND^*^	ND^*^	1480	0.57	nd	9.7	7.9	163	95.2	16.4	27.3	16.4	
		H1	11.4	0.53	nd	nd	0.05	0.13	1770	0.99	nd	8.3	7.8	191	48.4	25.2	32.1	25.2	
		H2	19.1	27.715	3852	3027	0.04	0.02	1385	2.19	445	nd2	7.8	152	65.1	49.9	13.6	49.9	
		H3	37.2	ND	2133	1890	0.11	0.11	1595	2.89	943	9	7.1	143	89.4	43.6	13.2	43.6	
		H4	36.1	0.05	1804	1665.5	0.10	0.11	1025	3.2	461	nd2	8.2	155	78.3	31.1	19.9	31.1	
		H5	18.6	0.05	569.3	439.7	0.11	0.12	725	4.58	nd	8.2	7.2	161	ND	14.7	18.3	14.7	
3	water		nd	nd	ND	ND	ND	ND	ND	ND	ND	ND	7.4	373	ND	ND	ND	NC
	sediments	H0	nd	0.07	nd	nd	0.11	0.12	1745	0.61	242	52.3	7.9	312	250	6.9	13.5	59	
		H1	6.8	0.05	nd	nd	0.11	0.07	1780	0.94	165	58.2	8	305	240	6.9	20.5	65.6	
		H2	33.3	1.07	2527	2039.4	0.03	0.02	1915	2.56	417	22.5	7.5	317	250	18.3	14.5	55.9	
		H3	50.1	9.22	3262.2	2779.9	0.03	0.1	1655	4.54	458	7.1	7.6	319	190	25.7	0.15	56.9	
		H4	20	1.16	2113	1408.5	0.03	0.09	ND	ND	ND	ND	7.8	308	ND	ND	ND	ND	
4	water		nd	nd	ND	ND	ND	ND	ND	ND	ND	ND	7.6	278	ND	ND	ND	NC
	sediments	H0	nd	0.05	nd	nd	ND^*^	ND^*^	1915	9.61	928	nd2	6.8	165	ND	25.7	20.4	25.7	
		H1	5.8	0.26	285.1	215	0.37	0.6	1895	13.72	nd	6.5	4	154	ND	39	18.4	39	
		H2	21	0.67	52.2	50	0.4	0.62	2610	12.27	nd	5.4	4	167	245	59	13.3	59	
		H3	25.3	3.895	5595	4767	0.47	0.79	1230	22.89	nd	nd2	3	149	195	91.9	16.3	91.9	
		H4	87.4	1.315	2051.2	1680	0.05	0.21	1805	14.9	nd	5.6	4.1	158	145	45.6	26.4	45.6	
		H5	13.7	2.51	790	729	0.09	0.1	1775	25.38	nd	nd2	6.7	164	130	55.7	19.4	55.7	
		H6	3.6	0.53	2233	2062	0.01	0.13	1315	4.09	nd	7.2	6.9	165	240	14.1	18.2	14.1	

ND, not determined; ND^*^, not calculated because C17 or C18 hydrocarbons were below to detection limit; nd, below detection limit (50 mg/kg); nd2, below detection limit (5 mg/kg); NC, the parameter does not correspond with the sample.

### Microbial Diversity

#### Diversity Indexes

Illumina sequencing results showed a total of 1,322,571 filtered sequences, ranging from 20,259 to 110,528 sequences per sample. The minor number of reads (20,259) was used to normalize the OTU table to the same sample depth. A total of 1,756 observed OTUs were generated after clustering at 97% similarity level. Setting this sample depth was enough to allow the coverage of all the samples, as observed by the plateau in the rarefaction curves (Supplementary Figure S1) and the calculated values of Good’s coverage index close to 1 (Table 2). Diversity indexes (richness Shannon, Simpson, and Chao) are presented in Table 2. Sediments of surface cores 1H0, 2H0, 3H0, and 4H0 showed the greatest diversity, with means of 8.56, 0.99, and 1,186 for Shannon, Simpson, and Chao indexes, respectively. Significant differences between surface and no surface core were found for Shannon and Chao indexes (*p* = 5.5 × 10^−7^; *F* = 37.46 and *p* = 5.5 × 10^−6^, *F* = 25.76). In contrast, the other samples ranged between 3.76–6.75, 0.77–0.98, and 352–970 for Shannon, Simpson, and Chao indexes, respectively, showing that the diversity in deepest cores was lower.

**Table 2:** Number of filtered sequences, diversity indexes (Shannon, Simpson, and Chao), richness related with observed operational taxonomic units (OTUs), and Nearest Sequenced Taxon Index (NSTI) scores in the sediment samples.

Samples	Number of reads after filtering	Good’s coverage index	Shannon index	Simpson index	Chao index	Richness	NSTI values
1H0	99,115	0.989	8.72	0.99	1,257	1,134	0.189
1H1	37,022	0.994	5.32	0.96	670	197	0.088
1H2	88,280	0.995	5.95	0.95	671	418	0.231
1H3	22,684	0.994	4.21	0.92	501	191	0.086
2H0	77,361	0.994	8.54	0.99	1,050	966	0.193
2H1	86,736	0.992	5.86	0.95	758	632	0.105
2H2	69,809	0.996	3.76	0.77	356	236	0.067
2H3	67,392	0.995	6.54	0.98	518	318	0.064
2H4	84,028	0.994	5.33	0.93	622	325	0.055
2H5	74,311	0.994	5.51	0.92	608	316	0.078
3H0	62,882	0.991	8.58	0.99	1,271	1,117	0.180
3H1	80,759	0.989	8.01	0.99	1,279	1,089	0.220
3H2	85,101	0.989	6.75	0.97	971	697	0.254
3H3	35,127	0.995	5.42	0.96	487	202	0.126
3H4	20,259	0.992	4.95	0.96	589	229	0.101
4H0	45,124	0.989	8.4	0.99	1,167	977	0.178
4H1	36,400	0.995	5.19	0.96	489	183	0.066
4H2	20,879	0.994	4.2	0.92	497	170	0.072
4H3	39,343	0.993	4.59	0.92	637	218	0.081
4H4	39,406	0.994	5.4	0.96	486	243	0.082
4H5	40,025	0.996	5.5	0.95	412	210	0.061
4H6	110,528	0.994	5.51	0.94	523	359	0.056

#### Beta Diversity

**Figure 2:**
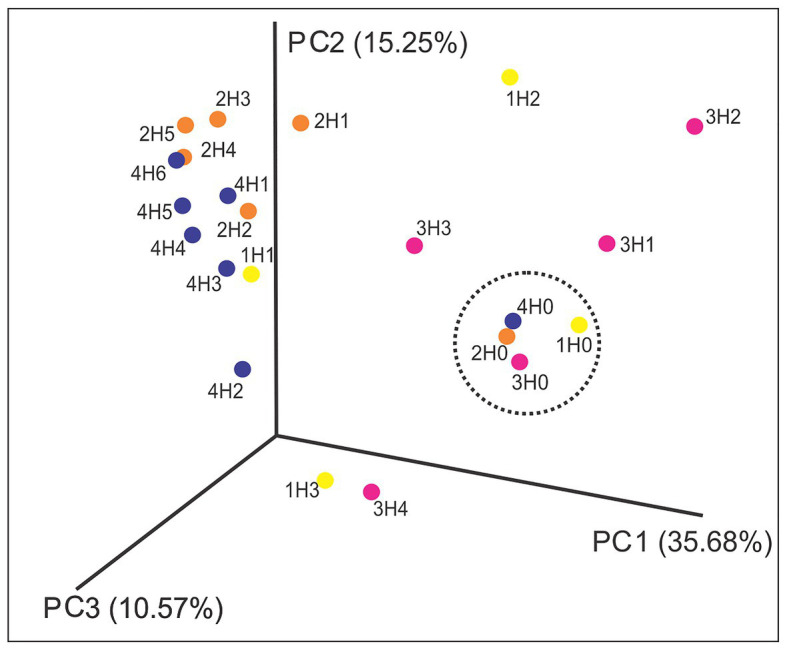
Principal coordinate analysis (PCoA) plot based on weighted UniFrac distance metric of samples (PERMANOVA test, 999 permutations). The black circle shows the clustering of surface sediment samples (1H0, 2H0, 3H0, and 4H0).

Principal coordinate analysis based on weighted UniFrac distance metric was performed in order to study the differences in bacterial community structure among samples. Figure 2 shows the dissimilarities of bacterial community structures across the samples. Variances between bacterial community structures were explained by the three axes: PCo1 (35.68%), PCo2 (15.25%), and PCo3 (10.57%). From the chart, it can be seen that the samples from the surface sediments (H0) were grouped together (marked in a black circle), evidencing the similarity in their bacterial community structures (PERMANOVA *p* = 0,001) and the differences in comparison with the others samples (PERMANOVA *p* = 0,001). The samples 1H1, 2H1, 2H2, 2H3, 2H4, 2H5, 4H1, 4H2, 4H3 4H4, 4H5, and 4H6 formed a second cluster in the Euclidean space, suggesting the presence of a similar community among them. The most divergent samples were 1H2, 1H3, 3H4, and 4H4.

#### Taxonomy Assignment

**Figure 3:**
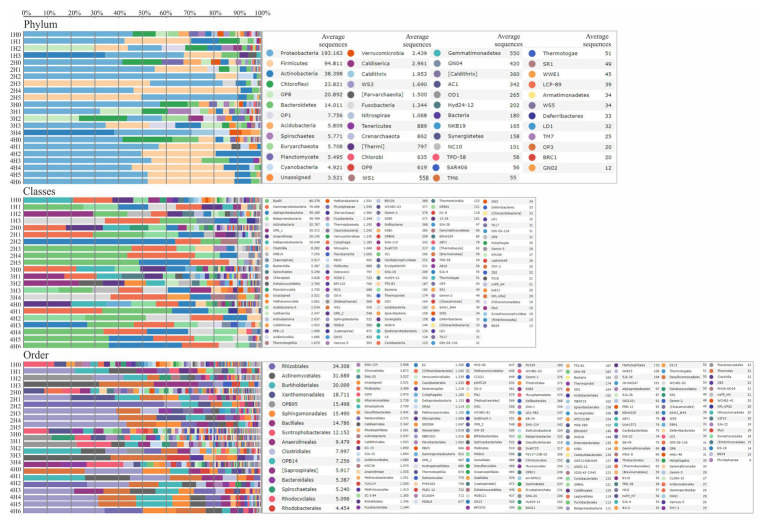
Taxonomy assignment in the samples under study showing the Relative Abundance at the Phylum, Class, and Order levels.

The bacterial communities of all the samples were almost dominated by the phylum Proteobacteria (40%) followed by: Firmicutes (18%), Actinobacteria (9%), OP8 (6%), and Chloroflexi (6%; Figure 3). The dominant phylum Proteobacteria consisted mainly of the classes: Gammaproteobacteria (36%), Alphaproteobacteria (20%), and Betaproteobacteria (17%). The phyla Firmicutes, Actinobacteria, and OP8, were dominated by the classes: Bacilli (16%), Actinobacteria (8%), and OP8_1 (6%), respectively (Figure 3). A remarkable predominance of the order Lactobacillales (13%) was found into Bacilli class, followed by Pseudomonadales (11%) belonging to Gammaproteobacteria class, Burkholderiales (6%) belonging to Betaproteobacteria class, Actinomycetales (8%) belonging to Actinobacteria class, and *OPB95* (8%) belonging to OP8_1 class (Figure 3). In relation to Archaea kingdom, the main classes present in sediments were Methanobacteria (0.4%) and Methanomicrobia (0.7%).

### Canonical Correspondence Analysis of the Differences in the Taxonomy Among Sediment Samples

**Figure 4:**
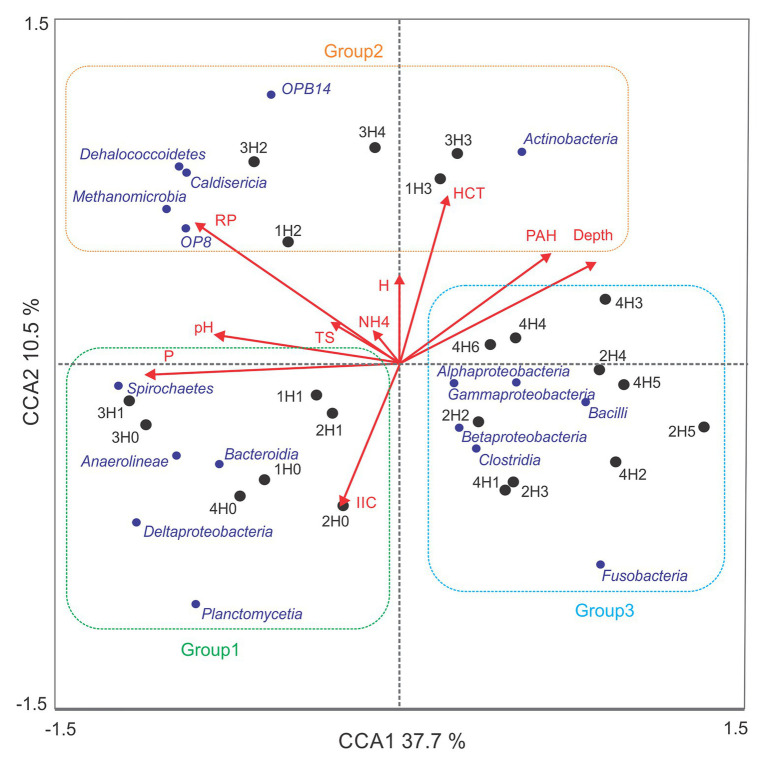
Canonical correspondence analysis of microbial community composition and environmental variables. Content of available phosphorus (P), redox potential (RP), pH, polycyclic aromatic hydrocarbons (PAH), total hydrocarbons (HCT), cation exchange capacity (IIC), total sulfide (TS), water content (H), and exchangeable ammonium (NH4). CCA1: first axis, CCA2: second axis. Samples belonging to group 1, group 2, and group 3 are gathered together in green, orange, and blue squares, respectively. Samples and classes are represented with black and blue dots, respectively.

The relationship between the structure of bacterial communities and physicochemical variables in sediment samples were studied with a CCA, which is presented in Figure 4. Eigenvalues for the first and second axes of the CCA explained a cumulative variance in the community composition of 48.2%, and a cumulative variance of the species–environment relationship of 78.1%. Montecarlo Test showed *p* = 0.0320 for the first canonical axis and *p* = 0.0230 of all canonical axes. Spatial distribution of the communities correlated significantly with depth (*p* = 0.0080, *F* = 3.388), available phosphorus (*p* = 0.0060, *F* = 4.905), redox potential (*p* = 0.0260, *F* = 3.671), and pH (*p* = 0.0280, *F* = 2.556). The variables: PAH (*p* = 0.0960, *F* = 2.114), THC (*p* = 0.2500, *F* = 1.296), cationic exchange capacity (*p* = 0.3280, *F* = 1.096), total sulfide (*p* = 0.4540, *F* = 0.891), water content (*p* = 0.7880, *F* = 0.506), and exchangeable ammonium (*p* = 0.891, *F* = 0.4840) were not significantly correlated with the composition of the bacterial community.

As shown in Figure 4, sediment samples were clustered in three different groups (groups 1–3). First, sediment samples from surface samples H0 (1H0, 2H0, 3H0, and 4H0) and H1 (1H1, 2H1, and 3H1), with the exception of 4H1, were clustered together in an area of the euclidean space represented by high available phosphorus, pH, and cation exchange capacity (group 1). The classes Deltaproteobacteria, Planctomycetia, Anaerolineae Spirochaetes, and Bacteroidia represent this group of samples. ANOVA and *post hoc* Tukey-Kramer statistical tests between the three groups showed that only Deltaproteobacteria, Planctomycetia, and Anaerolineae were significantly enhanced in group 1 (*p* < 0.05; Table 3B). Table 3C showed that Syntrophobacterales and Desulfobacterales, belonging to Deltaproteobacteria class, Anaerolineales and Methanomicrobiales, belonging to Anaerolinea and Methanomicrobia classes, respectively, were significantly enhanced in group 1.

**Table 3:** Relative abundance at phylum, class and order level and statistical analysis among sediment samples clustered in Group 1, 2, and 3.

A					
Kingdom	Phylum level	Samples			*p*
		Group 1	Group 2	Group 3	
Bacteria	Proteobacteria	45^a^	25^b^	50^a^	0.018
	Firmicutes	9^b^	10^b^	35^a^	7.23E-03
	Actinobacteria	4^a^	18^a^	6ª	>0.05
	Chloroflexi	12^a^	7^a,b^	0.2^b^	8.48E-03
	Planctomycetes	3^a^	0.9^a,b^	0.08^b^	0.035
	Spirochaetes	3^a^	2^a,b^	0.05^b^	>0.05
	OP8	6^a^	12^a^	0.1^a^	>0.05
	Latescibacteria (WS3)	2^a^	0.01^b^	0.006^b^	8.39-E3
	Acidobacteria	3^a^	0.8^a,b^	0.3^b^	0.032
Archaea	Euryarchaeota	2^a^	3^a^	0.02^a^	>0.05

B					
Phylum level	Class level	Samples			*p*
		Group 1	Group 2	Group 3	
Proteobacteria	Alphaproteobacteria	7^a^	6^a^	21ª	>0.05
	Betaproteobacteria	9^a^	5^a^	10ª	>0.05
	Gammaproteobacteria	17^a^	13^a^	19ª	>0.05
	Deltaproteobacteria	11^a^	2^b^	0.06^b^	2.49E-03
Firmicutes	Bacilli	8^b^	9^b^	32ª	4.29E-03
	Clostridia	1^a^	2ª	2ª	>0.05
Actinobacteria	Actinobacteria	2^a^	17^b^	6^a,b^	>0.05
Chloroflexi	Anaerolineae	11^a^	4^b^	0.09^b^	1.66E-03
Planctomycetes	Planctomycetia	2.5^a^	0.1^b^	0.06^b^	0.037
Spirochaetes	Spirochaetes	3^a^	2^a^	0.02ª	>0.05
OP8	OPB14	1^b^	5^a^	0.6^b^	0.034
	OP8_1	5^a^	1^a,b^	0.1^a,c^	0.018
Bacteroidetes	Bacteroidia	3^a^	0.6^a^	0.4^a^	>0.05
Euryarchaeota	Metanobacteria	0.6^a^	0.7^a^	0.004^a^	>0.05
	Metanomicrobia	0.6^a^	2^a^	0.02^a^	>0.05

C					
Class level	Order level	Samples			*p*
		Group 1	Group 2	Group 3	
Alphaproteobacteria	*Rhizobiales*	3^a^	4^a^	13^a^	>0.05
	*Sphingomonadales*	2^a,b^	0.5^b,c^	6^a^	>0.05
Betaproteobacteria	*Bulkhorderiales*	6^a^	4^a^	9^a^	>0.05
Gammaproteobacterias	*Pseudomonadales*	2^b^	12^a,b^	10^a^	0.029
	*Xanthomonadales*	8^a^	0.4^b,c^	4^a,b^	0.049
Deltaproteobacteria	*Syntrophobacterales*	7^a^	2^b^	0.04^b^	5.12E-03
	*Desulfobacterales*	1.7^a^	0.03^b^	0.007^b^	7.41E-03
Bacilli	*Lactobacillales*	7^b^	5^b^	27^a^	6.34E-03
Actinobacteria	*Actinomycetales*	2^a^	16^b^	6^a,b^	>0.05
Anaerolineae	*Anaerolineales*	6^a^	1^a,b^	0.05^b^	0.029
OP8_1	*OPB95*	2^a^	12^a,b^	0.09^a,c^	0.046
Bacteroidetes	*Bacteroidales*	3^a^	0.6^a^	0.4^a^	0.018
Euryarchaeota	*Methanomicrobiales*	0.3^a^	0.2^a,b^	0.02^b^	>0.05

Different letters (a,b,c) mean significant differences (*p* < 0.05) between groups. A number of taxonomic levels are shown: Kingdom and Phylum **(A)**, Phylum and Class **(B)**, and Class and Order **(C)**. The Statistical analysis was ANOVA and *post hoc* Tukey-Kramer tests, with Benjamini-Hochberg FDR multiple test correction and a *q* value filter of 0.05.

Samples 1H2, 1H3, 3H2, 3H3, and 3H4 were clustered in group 2, which correlated with high redox potential and THC. Dehalococcoidetes, Actinobacteria, Caldisericia, OP8, OPB14, and Methanomicrobia, were the most representative classes of this group. However, only the classes OPB14, with unclasiffied order_OPB14, and Actinobacteria, including members of Actynomycetales order, were found in significant (*p* < 0.05) and major relative abundances in this group of samples (Table 3C).

Group 3 gathered together, the deep samples of core 2 (2H2, 2H3, 2H4, and 2H5) and all hydrocarbon-contaminated samples of core 4 (4H1, 4H2, 4H3, 4H4, 4H5, and 4H6), characterized by an acid pH and by the fact of being taken from a point near to an urban discharge. Despite the fact that Alpha, Beta, Gammaproteobacteria, Bacilli, Fusobacteria, and Clostridia were the representative classes of this group, only Bacilli presented significantly higher relative abundance (*p* < 0.05), with almost exclusively members of Lactobacillales order. Among the orders affiliated to Gammaproteobacteria class, Pseudomonadales order was significantly more prevalent in groups 2 and 3 than in group 1.

### Predicted Functional Profiles and Metagenome Contribution of the Bacterial Communities

In order to determine whether sediment samples were tractable for PICRUSt prediction, NSTI values were calculated (Table 2). As described by Langille et al. (2013), the accuracy of PICRUSt prediction in general decreases with increasing NSTI values. For most samples, NSTI values were below 0.18, which agreed with the mid-range values calculated in soils by Langille et al. (2013). Conversely, NSTI values were greater (0.18–0.25) for 1H0, 1H2, 2H0, 3H0, 3H1, 3H2, and 4H0 samples. It is expected that these samples contain much unexplored diversity, and therefore PICRUSt results should be interpreted with caution.

#### Functional Prediction

**Figure 5:**
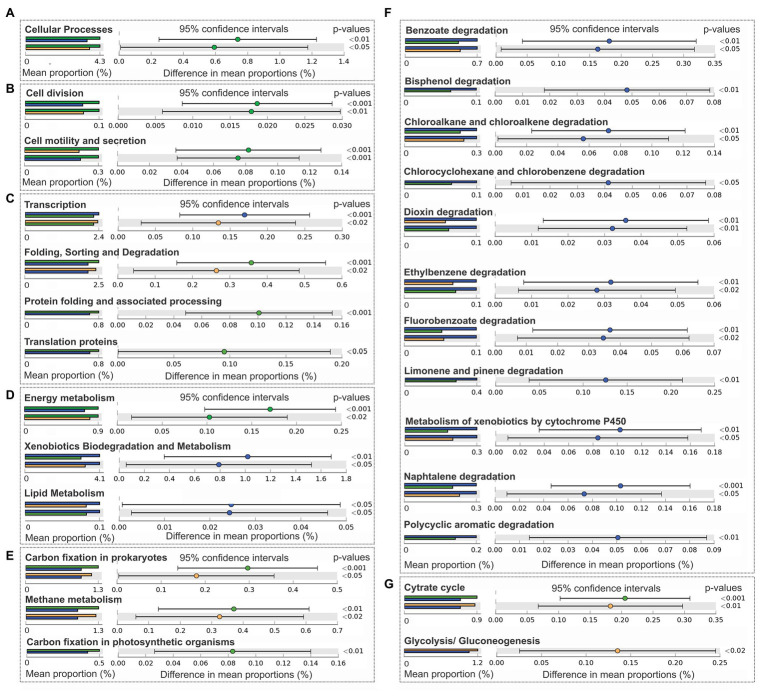
Comparison of predictive functions at Level 1, Level 2, and Level 3 of KEGG categories (PICRUSt) between bacterial communities from Group 1 (green), Group 2 (orange), and Group 3 (blue). **(A)** Cellular processes (Level 1). **(B)** Subcategories cellular processes and signaling: cell division and cell motility and secretion (Level 2). **(C)** Genetic information processing: transcription, folding sorting and degradation, Protein folding and associated processing, and translation proteins and metabolism (Level 2). **(D)** Metabolism: energy metabolism, xenobiotic degradation and metabolism, and lipid metabolism (Level 2). **(E)** Energy metabolism: carbon fixation by prokaryotes, methane metabolism, and carbon fixation in photosynthetic organisms (Level 3). **(F)** Subcategories xenobiotics degradation and metabolism: benzoate degradation, bisphenol degradation, chloroalkane and chloroalkene degradation, chlorocyclohexane and chlorobenzene degradation, dioxin degradation, ethylbenzene degradation, fluorobenzoate degradation, limonene and pinene degradation, metabolism by xenobiotics by cytochrome 450, naphthalene degradation, and polycyclic aromatic degradation. **(G)** Carbohydrate metabolism: cytrate cycle, glycolysis, and gluconeogenesis (Level 3).

Significant results of PICRUSt functional prediction are summarized in Figure 5. As indicated by the comparison between groups, sediment samples clustered in group 1 showed a significant enrichment in the number of sequences included in the category of cellular processes (Figure 5A), and in the two subcategories of cell division, cell motility, and secretion (Figure 5B), protein folding and associated processing, translation, folding sorting and degradation (Figure 5C), and energy metabolism (Figure 5D), in relation with group 2 and 3. In this group also prevailed the pathways involved in: carbon fixation in prokaryotes and in photosynthetic organisms (Figure 5F), methane metabolism (Figure 5E), and citrate cycle (Figure 5G). Glycolysis and gluconeogenesis were enriched in sediments of group 2 (Figure 5G) in comparison with samples from groups 1 and 3. Sediments of group 3 were significantly enriched in lipid metabolism and xenobiotics biodegradation and metabolism subcategories (Figure 5D), being the degradation pathways of benzoate, bisphenol, chlorocyclohexane and chlorobenzene, chloroalkane and chloroalkene, dioxin, ethylbenzene, fluorobenzoate, limonene and pinene, naphthalene, polycyclic aromatic hydrocarbons, and the metabolism of xenobiotics by cytocrome P450 enhanced in group 3, in comparison with group 1 and 2 (Figure 5F).

#### Metagenome Contributions

Table 4 provides an overview of several KEGG orthologs (K0s), which were involved and significantly increased in the most relevant metabolic pathways of every group. In addition, Table 4 also shows the number of sequences predicted in most relevant classes for each K0.

**Table 4:** Kyoto Encyclopedia of Genes and Genomes (KEGG) orthologs (K0) involved in three pathways: (i) carbon fixation by photosynthetic microorganism, (ii) xenobiotics biodegradation and metabolism, and (iii) stress response and resistance to heavy metals, and its contribution by bacteria (class taxonomic level).

Protein name	K0	EC number	Gene	Contribution by class	Count contributed by OTU		
					Group 1	Group 2	Group 3
**Carbon fixation by photosynthetic microorganisms**							
ribulose-bisphosphate carboxylase large chain	K01601	4.1.1.39	*rbcL*	Alphaproteobacteria	2,301	1,233	6,538
				Anaerolineae	5,058	817	66
				Betaproteobacteria	3,071	713	1,678
				Gammaproteobacteria	7,305	510	927
				Total	18840^a^	4743^b^	9916^b^
carbon monoxide dehydrogenase/acetyl-CoA synthase	K00198	1.2.7.4	*cooS*	Deltaproteobacteria	16,982	2,257	111
				Total	20340^a^	7449^a,b^	1409^b^
pyruvate-ferredoxin/flavodoxin oxidoreductase	K03737	1.2.7.1/1.2.7	*nifJ*	Anaerolineae	5,058	868	15
				Deltaproteobacteria	4,008	499	14
				Total	15365^a^	3487^b^	4260^b^
**Xenobiotics biodegradation and metabolism**							
catechol 1.2-dioxygenase	K03381	1.13.11.1	*catA*	Alphaproteobacteria	1753	1,221	6,327
				Betaproteobacteria	975	29	2,698
				Gammaproteobacteria	374	568	3,296
				Total	4051^b^	2701^b^	13105^a^
glutathione S-transferase	K00799	2.5.1.18	*gst*	Alphaproteobacteria	21,106	4,053	111,801
				Betaproteobacteria	21,109	2,355	11,245
				Gammaproteobacteria	28,203	2,661	20,240
				Total	72582^b^	9309^b^	148445^a^
3-oxoadipate CoA-transferase	K01031	2.8.3.6	*pcaI*	Actinobacteria	464	2,482	570
				Alphaproteobacteria	457	1,190	2,743
				Betaproteobacteria	1957	5,147	2,448
				Gammaproteobacteria	464	2,482	570
				Total	3773^b^	3797^a,b^	13831^a^
2-keto-4-pentenoate hydratase	K02554	4.2.1.80	*mhpD*	Alphaproteobacteria	1,309	144	2,937
				Betaproteobacteria	903	40	105
				Gammaproteobacteria	673	181	1,274
				Total	3405^a,b^	525^b^	4901^a^
lysophospholipase	K01048	3.1.1.5	*pldB*	Actinobacteria	188	178	1783
				Alphaproteobacteria	3,204	1,464	12,685
				Gammaproteobacteria	651	10	711
				Total	5854^b^	2034^b^	16206^a^
esterase/lipase	K01066	3.1.1.-	*aes*	Alphaproteobacteria	2,867	3,036	19,688
				Betaproteobacteria	2,192	1,338	5,368
				Gammaproteobacteria	1933	5,019	10,345
				Total	9495^b^	11003^a,b^	38481^a^
alkanesulfonate monooxygenase	K04091	1.14.14.5	*ssuD*	Alphaproteobacteria	518	2,187	3,068
				Alphaproteobacteria	4,736	3,533	22,380
				Betaproteobacteria	4,519	2,515	9,806
				Gammaproteobacteria	2,754	4,403	14,760
				Total	15041^b^	13925^a,b^	50948^a^
**Stress response and resistance to heavy metals**							
heat shock protein HspQ	K11940	–	*HspQ*	Alphaproteobacteria	3,591	1,550	14,572
				Total	3954^b^	7166^b^	14756^a^
Redox-sensitive transcriptional activator SoxR	K13639	–	*soxR*	Alphaproteobacteria	1746	470	9,685
				Betaproteobacteria	1,356	1892	4,432
				Gammaproteobacteria	1,081	2,545	6,274
				Total	5165^b^	5801^a,b^	22770^a^
heat shock response in Gram-positive. ctsR	K03708	–	*ctsR*	Bacilli	2066	1,463	11,857
				Total	2498^b^	4635^b^	12949^a^
starvation-inducible DNA-binding protein	K04047	–	*dps*	Alphaproteobacteria	3,520	2088	22,330
				Bacilli	2,140	2,243	12,812
				Betaproteobacteria	4,364	1813	6,209
				Gammaproteobacteria	6,397	2,700	8,574
				Total	27527^b^	16001^b^	57347^a^
osmoprotectant transport system substrate-binding protein	K05845	–	*opuC*	Alphaproteobacteria	1,207	746	10,433
				Bacilli	1,526	1,110	10,006
				Total	6845^b^	8586^b^	30942^a^
osmoprotectant transport system permease protein	K05846	–	*opuBD*	Alphaproteobacteria	2,440	1857	21,092
				Bacilli	3,927	1,586	24,665
				Total	25978^b^	24248^b^	68948^a^
osmoprotectant transport system ATP-binding protein	K05847	7.6.2.9	*opuA*	Alphaproteobacteria	1,399	1,168	11,785
				Bacilli	2,331	1702	13,174
				Total	13460^b^	13066^b^	40900^a^
osmolarity sensor histidine kinase	K07638	–	*envZ*	Alphaproteobacteria	2,409	8,918	4,385
				Betaproteobacteria	6,507	4,068	8,161
				Gammaproteobacteria	3,963	6,213	7,243
				Total	12655^b^	6264^b^	27614^a^
general stress protein 13	K07570	–	*GSP13*	Bacilli	2,166	1,471	12,536
				Total	2166^b^	1471^b^	12536^a^
arsenical resistance protein	K11811	–	*arsH*	Alphaproteobacteria	2,273	1,046	12,840
				Betaproteobacteria	1,528	869	4,482
				Gammaproteobacteria	2,934	2,657	8,183
				Total	6765^b^	4572^b^	25529^a^
arsenate reductase	K00537	1.20.4.1	*arsC*	Alphaproteobacteria	5,982	2,885	27,026
				Bacilli	16,358	55,052	123,776
				Betaproteobacteria	4,356	2,550	9,404
				Gammaproteobacteria	9,755	5,416	13,373
				Total	30921^b^	16164^b^	88211^a^
copper homeostasis protein	K06201	–	*cutC*	Alphaproteobacteria	292	376	7,826
				Bacilli	2081	1,179	12,124
				Gammaproteobacteria	1796	91	2,223
				Total	9912^b^	4513^b^	26039^a^

The table shows only bacterial classes and K0 with significant differences between Groups (ANOVA and *post hoc* Tukey-Kramer tests, with Benjamini-Hochberg FDR multiple test correction and a *q* value filter of 0.05). Different letters (a,b) mean significant differences (p < 0.05) between groups.

Regarding the pathway of carbon fixation by photosynthetic microorganisms, the ribulose-bisphosphate carboxylase large chain gene (EC:4.1.1.39, K01601), contributed by the classes Gammaproteobacteria, Anaerolineae, Alphaproteobacteria, and Betaproteobacteria, was significantly enriched in group 1. Involved in the carbon fixation pathway in prokaryotes, the gene encoding the carbon monoxide dehydrogenase/Acetyl-CoA synthase (EC:1.2.7.4, K00198) was almost exclusively contributed by the order Syntrophobacterales (data not shown), which belongs to Deltaproteobacteria. The pyruvate-ferredoxin/flavodoxin oxidoreductase gene (EC:1.2.7.1 1.2.7.-, K03737) was significantly enhanced in the group 1 and provided by Deltaproteobacteria and Anaerolineae (Table 4).

Regarding xenobiotics biodegradation and metabolism, Alphaproteobacteria, Betaproteobacteria, and Gammaproteobacteria (Table 4) were the classes which major proportions of sequences encoding the degrading genes: catechol 1,2-dioxygenase (K03381), glutathione S-transferase (K00799), 3-oxoadipate CoA-transferase (K01031, K01032), and 2-keto-4-pentenoate hydratase (K02554). Genes involved in lipid metabolism were: lysophospholipase (K01048), esterase/lipase (K01066), and alkanesulfonate monooxygenase (K04091). All these genes were significantly enhanced in group 3.

A number of K0 related with a stress response and heavy metal resistance were also significantly enhanced in group 3: heat shock protein HspQ (K11940), MerR family transcriptional regulator, redox-sensitive transcriptional activator SoxR (K13639), transcriptional regulator of stress and heat shock response ctsR (K03708), starvation-inducible DNA-binding protein (K04047), osmoprotectant transport system substrate-binding protein (K05845, K05846, and K05847), osmolarity sensor histidine kinase (K07638), general stress protein 13 (K07570), arsenical resistance protein (K11811), arsenate reductase (K00537), and copper homeostasis protein (K06201). Metagenome contribution showed that the classes with major number of sequences of these K0 were: Bacilli, Alphaproteobacteria, Betaproteobacteria, and Gammaproteobacteria (Table 4). In particular, the K0 of general stress protein 13 (GST13) and heat shock response were enhanced in Lactobacillales and the K0 of osmoprotectant transport system substrate-binding protein in Lactobacillales and Rhizobiales orders.

## Discussion

Monitored natural recovery is a sustainable remedy approach highly site-specific that, and, in many cases, may require years or decades to establish the amount of risk reduction desired by stakeholders (Magar and Wenning, 2006). For this reason, MNR combined with a thin-layer cap of clean sand or clean sediment over the contaminated sediment is generally selected. The capping to exceed the depth of bioactive zone, typically 5–15 cm, so that immediate risk reduction can be achieved (Fetters et al., 2020). The chemical analyses in the present work showed that the hydrocarbon concentration was below the detection limit (< 50 mg/kg) both in water and surface sediments samples. These results are likely to be related to a capping process, in which natural sedimentation permitted the physical isolation of the hydrocarbons. Interestingly this allowed the constitution of an effective hydrocarbon primary source control barrier that led to pollutant-risk reduction. In addition, the ratios nC17/pristane and nC18/phytane were used as indicators of biodegradation (Christensen and Larsen, 1993; Companioni-Damas et al., 2011). Isoprenoids (i.e., pristane, phytane) present a greater resistance to biodegradation than the n-alkanes. The fact that such ratios were generally less than one was indicative of the occurrence of advanced biodegradation process in non-surface sediments, suggesting that natural attenuation could have happened after industrial source control (20 years ago).

The bacterial diversity results showed very similar community structures among surface sediments, with a greater and distinct diversity than that observed for the non-surface and contaminated sediments (Figure 3). These results are in agreement with a previous study indicating that due to intensive resuspension events, the bottom bathymetry and the patchiness of submerged vegetation, the bacterial community composition in the upper 2–5 cm tends to remain homogenous (Tšertova et al., 2013). Furthermore, different reports of biomass and enzymatic activity measurements showed that bacterial abundance and total activity are generally higher near the sediment surface and tend to decrease with depth (Haglund et al., 2003; Wurzbacher et al., 2017). These observations are probably related to the process of natural sediment formation, in which a proportion of newly settled organic matter is rapidly recycled in the surface by biological activity and subsequently transformed into secondary compounds. Consequently, vertical gradients of respiratory electron acceptors are generated (Froelich et al., 1979), being organic matter and electron acceptors the main processes that structure the microbial community (Wurzbacher et al., 2017). Multivariable analysis was performed in order to put in evidence which is the main variables that structure the community composition in the present sediment samples (Figure 4). The analysis showed yet again similarities among surface samples (Group 1), whereas the bacterial community of the non-surface sediments samples showed to be more heterogeneous, forming two separate clusters (Groups 2 and 3). Depth, available phosphorous, redox potential, and pH, but not the hydrocarbon concentration, were the environmental variables that differentiated the community composition. These results are in agreement with previous observations, which showed that the selection pressure exerted by natural filter in sedimentary coastal environments is greater than that exerted by hydrocarbons (Jeanbille et al., 2016).

*In silico* metagenomes obtained in the present work further support the idea of a superficial enhanced metabolic activity, as demonstrated by the enrichment of cellular processes, like cell division, cell motility, and genetic information processing in surface sediments (Figure 5). The major contribution to the diversity and extensive metabolic capacity of the surface sediments would be delivered by the presence of the orders: Syntrophobacterales, Desulfobacterales, Anaerolineales, and Methanomicrobiales (affiliated to Deltaproteobacteria, Planctomycetia, Anaerolineae, and Methanomicrobia classes, respectively; Figures 3, 4; Table 3). The enhanced carbon fixation pathways in surface samples suggest the prevalence of autotrophic processes, which could be achieved through three different pathways of carbon assimilation by microbial communities. First, Deltaproteobacteria and Anaerolineae probably perform the carbon fixation through the reductive citric acid cycle (Arnon-Buchanan cycle), since it has been predicted that these classes possess genes that encode the enzyme pyruvate-ferredoxin/flavodoxin oxidoreductase (K03737; Table 4). Second, Syntrophobacterales order has been demonstrated to possess the ability to fix carbon through the reductive acetyl-CoA pathway (Wood-Ljungdahl pathway; related to K00198: carbon monoxide dehydrogenase/acetyl-CoA synthase). Finally, the classes Gammaproteobacteria, Alphaproteobacteria, and Anaerolineae could contribute to carbon fixation by the Calvin cycle as it has been predicted that these classes own genes that encode the large chain of the enzyme ribulose-bisphosphate carboxylase (EC:4.1.1.39, K01601; Table 4).

Consistent with the literature (Grabowski et al., 2005), heterotrophic and fermentative members of Deltaproteobacteria co-occurred with hydrogenotrophic methanogens (H_2_ and CO_2_ consumers) in surface sediments (Table 3C). The presence of Syntrophobacterales and Desulfobacterales, together with Methanomicrobiales and the consequent enrichment in methane metabolism pathways (Figure 5E), suggests that methanogenesis could be performed by these taxa. Probably, this pathway occurs in surface sediments reducing CO_2_ by formylmethanofuran dehydrogenase (EC:1.2.7.12, subunit A, K00200; Table 4). Particularly, members of the orders Desulfobacterales and Syntrophobacterales are sulfate-reducing bacteria able to grow as syntrophs in cooperation with methanogens in anoxic sediments (Morris et al., 2013). The decline of the relative abundance of Desulfobacterales with sample depth could be related to an important deterioration of “ecosystem health” (Jeanbille et al., 2016). The presence of autotrophic and syntrophic microorganisms in surface samples, involved in natural processes and commonly found in anoxic sediments suggest the establishment of a microbial community with the ability to restructure the environmental functions of the site.

Some authors have related diversity reduction to the persistence and dominance of certain taxa whose relative abundance increases with sediment depth. These persistent taxa have shown a number of advantageous features like metabolic versatility, mechanisms of resistance to environmental factors, and/or adaptations to energy limitations (e.g., substrate fermentation, carbon storage capacity, DNA repair mechanisms, sporulation ability, high ATP synthesis efficiency, and reduced genome size; Petro et al., 2017; Starnawski et al., 2017; Rissanen et al., 2019). In the present work, according to the metagenomic prediction and its abundance in acid-pH sediment samples, Bacilli could be a likely candidate for a persistent taxon (Tables 3, 4; Figure 5). Members of this class showed the genetic capacity to respond to different kinds of environmental stresses (e.g., oxidative, energy, and acid-pH stress), inducing the expression of an alternative sigma factor (*σ*^B^) GST13 (K07550), which activates a large set of general stress genes, thereby conferring broad resistance in natural environments (Petersohn et al., 2001; Price et al., 2001). The heat shock response in Gram-positive bacteria is regulated by *ctsR* gene (K03708) and under osmotic stress, the presence of ABC transporter Opu A, B, C (K05845/6/7) could confer to Bacilli the capacity to resist the acid pH and hostile environment of deep sediments (Table 4). Besides, the presence of number of K0s that respond to metal ion stress were also found in Bacilli and Proteobacteria (*ars* genes K11811, K00537, and K06201). In agreement with this, genes implicated in the cross capacity to resist both acid pH and lead have also been reported for Lactobacillales (Giri et al., 2019). Therefore, all these genes may allow Bacilli to withstand heavy metal, acid-pH, and osmotic stress, characteristics that are actually present in deeper sediments from cores 2 and 4 (Table 4; Figure 5). Notably, in deep samples from cores 1 and 3, which are under the influence of a positive redox potential and a high hydrocarbon concentration, the presence of OPB14 class and sequences of acetyl-CoA synthase (K00198) involved in Wood-Ljungdahl pathway were enriched (Table 4). Although, due to the lack of subsurface cultured representatives, it is difficult to link the phylogenetic patterns in deep sediments, it is known that there are many groups that are capable of growing fermentatively or by acetogenesis in deep layers (Petro et al., 2017). In contrast to surface samples, the taxonomic contribution of acetyl-CoA synthase suggests that the class OPB14 could be responsible for carbon fixation in the non-surface samples, clustered in Group 2 (Table 4).

Metabolic pathways for hydrocarbon degradation under anoxic conditions are fairly well understood (Sierra-Garcia and Oliveira, 2013). Phylogeny and metagenome prediction suggest that degrading bacteria belonging to Alphaproteobacteria, Betaproteobacteria, Gammaproteobacteria, and Actinomyces classes could be responsible for the potential xenobiotic degradation in deeper sediments (Table 4). In spite of the anoxic nature of sediments, these classes contributed mainly with genes linked to aerobic degradation of aromatics [e.g., glutathione S-transferase (K00799), 3-oxoadipate-succinyl-coenzyme A-CoA transferase (K01031), 3-oxoadipyl-CoA thiolase (K01032), catechol 1,2-dioxygenase (K03381), and NADP-dependent aldehyde dehydrogenase (K14519)] and aliphatic hydrocarbons [esterase/lipase (K01066), lysophospholipase (K01048), and alkanemonosulfonate monooxigenase (K04091); Table 4]. In freshwater sediments, the oxygen is often limited to the surface and decreases within the first few millimeter or centimeter down the sediment core (Barker Jørgensen and Peter Revsbech, 1985). Below that depth, other electron acceptors, such as nitrate, iron, or sulfate become energetically favorable for the microorganisms (Rivett et al., 2008). The degradation of alkanes and aromatic hydrocarbons under nitrate-reducing conditions has been detected in *in vitro* assays as phenol-degrading enrichment cultures (Baek et al., 2003), in microcosms made with contaminated subsurface aquifer sediments (Wang et al., 2012a) and in the environment (Martirani-Von Abercron et al., 2016). In accordance with the present results, the bacterial diversity in the above-mentioned works was limited to subclasses of Proteobacteria, and some Gram positive bacteria (Baek et al., 2003; Wang et al., 2012b; Martirani-Von Abercron et al., 2016). In particular, members of Pseudomonadales have been demonstrated to degrade hydrocarbons using nitrate as electron donor (Mcnally et al., 1999), suggesting that the increment of this family in deeper sediments could be associated with this metabolic capacity (Table 3). Recently, some authors have described that during nitrate respiration some microorganisms can produce intracellular oxygen through a putative nitric oxide dismutase advantageous for aerobic pathways in anoxic environments (Zedelius et al., 2011; Ettwig et al., 2012). Likewise, other authors could quantify a high proportion of the corresponding *nod* gene in the environment (Zhu et al., 2017), suggesting that the occurrence of aerobic degradation pathways under anoxic, denitrifying conditions should not be excluded.

The characterization of sediments from a historically anthropized tributary drainage channel of Río de La Plata by environmental measures, metabarcoding, and functional prediction approaches, allowed to assess how the microbial activity would contribute to the recovery of the site. The evidence from this study suggests the formation of a natural and uncontaminated sediment cap, which allowed the exclusion of hydrocarbons from the water, thus possibly preventing pollutants entering the food chain. In addition, the findings of this research indicate the presence of microorganisms related with “ecosystem health” in natural and uncontaminated cap (H0); whereas, in deeper and contaminated sediments, hydrocarbon degradation pathways were predicted.

Overall, this study strengthens the idea that the use of metabarcoding and bioinformatics tools to assess the structure, diversity, and function of sediment bacterial communities in association with a deep analyses of site physicochemical and geochemical characteristics, proved to be useful to elucidate the possible contribution of biological processes to hydrocarbon degradation and site recovery. As MNR relies on natural biodegradation processes, this study could support the selection of MNR as a recovery strategy of the present anthropized freshwater course. This is the first time that such an approach is undertaken in the region.

Finally, this study intends to encourage the incorporation of metabarcoding techniques and bioinformatics approaches to provide new insights for MNR assessment.

## Data Availability Statement

The datasets presented in this study can be found in online repositories. The names of the repository/repositories and accession number(s) can be found in the article/Supplementary Material.

## Author Contributions

LM conceived and designed the experiments, analyzed the data and performed the computational analysis, contributed reagents, and materials, prepared figures and tables, and wrote and reviewed drafts and the final version of the paper. VS performed the laboratory analysis and sample extraction, contributed with reagents, materials, and analysis tools, prepared figures and tables, and reviewed drafts and the final version of the paper. AA analyzed DNA sequencing data, performed the computational analysis (i.e., 16S Bacteria and Archaea Standard Operating Procedure, PICRUSt pipeline), and reviewed (e.g., English language editing) drafts and the final version of the paper. BC analyzed the data and reviewed drafts of the paper. CL conducted environmental sampling, analyzed the data, and reviewed drafts of the paper. NV conceived the experiments and reviewed drafts of the paper. PM conceived the experiments and reviewed drafts of the paper. MO conceived and designed the experiments. MP conceived and designed the experiments, analyzed the data, and wrote and reviewed drafts of the paper. IM conceived and designed the experiments, analyzed the data, and wrote and reviewed drafts and the final version of the paper. All authors contributed to the article and approved the submitted version.

### Conflict of Interest

MO and NV are employed by the company YPF Tecnología. PM is employed by the company YPF S.A.

The remaining authors declare that the research was conducted in the absence of any commercial or financial relationships that could be considered as a potential conflict of interest.

## Footnotes

^1^https://www.ncbi.nlm.nih.gov/

^2^https://scistatcalc.blogspot.com/2013/11/one-way-anova-test-calculator-in.html#

^3^http://kiwi.cs.dal.ca/Software/STAMP

^4^http://www.genome.jp/kegg/
