# Supplementary material for: Assessment of Biological Contribution to Natural Recovery of Anthropized Freshwater Sediments From Argentina: Autochthonous Microbiome Structure and Functional Prediction
Source: Front Microbiol. 2021 Mar 5;12:601705. doi: 10.3389/fmicb.2021.601705 (PMC8059475; doi:10.3389/fmicb.2021.601705)
Supplement: Supplementary file 1 [file Data_Sheet_1.docx]

Supplementary Material

# Supplementary Figures and Tables

**1.1 Supplementary Tables**

| Core ID | Core length  (cm) | Number of horizons | Name of each horizon | Depth (cm) |
| --- | --- | --- | --- | --- |
| **1** | 177 | 4 | H0 | 13.2 |
|  |  |  | H1 | 21.2 |
|  |  |  | H2 | 147 |
|  |  |  | H3 | 179 |
| **2** | 194 | 6 | H0 | 13.5 |
|  |  |  | H1 | 19 |
|  |  |  | H2 | 63 |
|  |  |  | H3 | 126 |
|  |  |  | H4 | 177 |
|  |  |  | H5 | 194 |
| **3** | 74.5 | 5 | H0 | 7 |
|  |  |  | H1 | 27.2 |
|  |  |  | H2 | 60 |
|  |  |  | H3 | 70 |
|  |  |  | H4 | 74 |
| **4** | 104.5 | 7 | H0 | 9 |
|  |  |  | H1 | 22 |
|  |  |  | H2 | 30 |
|  |  |  | H3 | 57 |
|  |  |  | H4 | 68.6 |
|  |  |  | H5 | 91 |
|  |  |  | H6 | 131 |

**Supplementary Table S1:** Samples characteristics and number of subsamples (lithological units/horizons).

**1.2 Supplementary Figures**


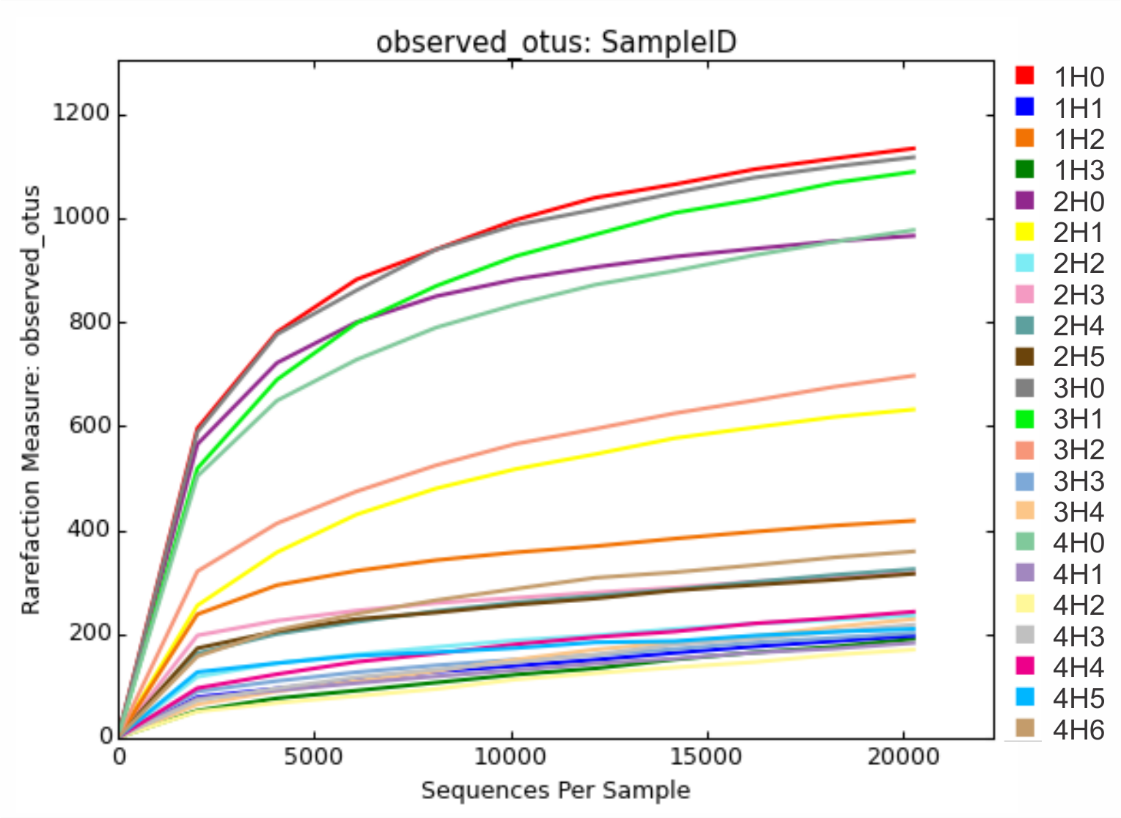


**Supplementary Figure S1:** Rarefaction curves of the sediment samples.
